# Strain Differences Determine the Suitability of Animal Models for Noninvasive *In Vivo* Beta Cell Mass Determination with Radiolabeled Exendin

**DOI:** 10.1007/s11307-016-0936-y

**Published:** 2016-02-17

**Authors:** Stefanie M. A. Willekens, Lieke Joosten, Otto C. Boerman, Alexander Balhuizen, Decio L. Eizirik, Martin Gotthardt, Maarten Brom

**Affiliations:** 1 Department of Radiology and Nuclear Medicine, Radboud University Medical Center, PO BOX 9101, 6500 HB Nijmegen, The Netherlands; 2 ULB Center for Diabetes Research, Université Libre de Bruxelles (ULB), Brussels, Belgium

**Keywords:** Diabetes, Beta cell mass, Receptor targeting, GLP-1R, Exendin

## Abstract

**Purpose:**

Noninvasive beta cell mass (BCM) quantification is a crucial tool to understand diabetes development and progression. [^111^In]exendin is a promising agent for *in vivo* beta cell imaging, but tracer testing has been hampered by the lack of well-defined rodent models.

**Procedures:**

Biodistribution and pancreatic uptake of [^111^In]exendin were compared in rats and mice. In selected models, the amount of [^111^In]exendin accumulation in the pancreas and other organs was determined using a model of alloxan-induced beta cell loss. GLP-1R expression levels were analyzed by RT-PCR and immunohistochemistry.

**Results:**

Namely Brown Norway rats showed beta-cell-specific tracer accumulation and favorable pancreas-to-background ratios for noninvasive BCM determination. Mice displayed receptor-mediated [^111^In]exendin uptake in endocrine and exocrine pancreas, in spite of very low GLP-1R expression in exocrine tissue.

**Conclusions:**

Rats display better characteristics for *in vivo* BCM determination than mice and are suggested as a more adequate model for humans.

**Electronic supplementary material:**

The online version of this article (doi:10.1007/s11307-016-0936-y) contains supplementary material, which is available to authorized users.

## Introduction

Diabetes mellitus is characterized by defective glucose homeostasis and consequent chronic hyperglycemia. Maintenance of glycemic control by daily insulin injections or anti-diabetogenic drugs is difficult, and patients affected by both type 1 and type 2 diabetes are at risk for severe complications, such as cardiovascular disease, blindness, and kidney failure, increasing their risk for premature death. Changes in beta cell mass (BCM) occur in both forms of diabetes [1–3], but the mechanisms and evolution underlying these BCM changes are poorly understood [4, 5].

Beta cell function is usually monitored by measurements of insulin secretion, such as insulin and C-peptide, analyzed in parallel to glucose levels. However, it was shown that beta cell function and BCM do not diminish equally during disease progression [5, 6], indicating that beta cell function is not an adequate measure for BCM. A reliable and reproducible method to monitor BCM dynamics over time, such as longitudinal quantitative imaging, could unravel the importance of BCM during the course of diabetes.

During the past decade, major efforts have been made to image BCM *in vivo* in animal models using a variety of imaging methodologies [7], such as near infrared optical projection tomography (OPT) [8], bioluminescence [9], or magnetic resonance imaging (MRI) [10–12]. While MRI offers the highest resolution (still not allowing resolution of single islets *in vivo*), nuclear medicine imaging modalities, such as single photon emission computed tomography (SPECT) and positron emission tomography (PET), have the potential to reach sufficient sensitivity to detect small numbers of beta cells when targeted with a radiolabeled beta-cell-specific tracer. Importantly, the first clinical trial results show that SPECT and PET are the most likely approaches to reach human translation at this point in time [13–15].

A variety of antibodies, such as IC2 [16]; small molecules, such as 2-deoxy-2-[^18^F]fluoro-D-glucose [17]; neurotransmitter precursors, such as [^11^C]5-hydroxytryptophan ([^11^C]5-HTP) [18]; organic compounds, such as dihydrotetrabenazine (DTBZ) targeting the vesicular monoamine transporter 2 (VMAT2) [19]; and peptides, such as exendin targeting the glucagon-like peptide 1 (GLP-1) receptor [13] have been evaluated as tracers for BCM determination. Very recent findings indicate that [^11^C]5-HTP can successfully discriminate between rats with severe or moderate beta cell loss [15, 18], while radiolabeled exendin, a stable GLP-1R agonist specifically targeting the pancreatic beta cells [20], was shown to successfully detect small changes in BCM in a preclinical model for beta cell loss [14]. In order to further validate the use of GLP-1R radionuclide imaging to evaluate the dynamics of BCM changes in diabetes, further studies in animal models are required.

A variety of preclinical models have been used to investigate BCM dynamics, but the results obtained showed differences in biodistribution and in tracer uptake in relation to BCM [14, 21–24]. We hypothesize that the observed differences in these studies can be related to the use of animal models. Therefore, in the present study, we aimed to evaluate the differences between various animal models to select the most suitable model for preclinical, noninvasive BCM determination using [^111^In]exendin. Ideally, this optimal model should allow accurate, quantitative BCM determination and its features should reflect the human situation. We compared the biodistribution and pancreatic uptake of [^111^In]exendin in various rat and mouse strains and compared their specific GLP-1R expression pattern. Finally, we determined the specificity of [^111^In]exendin accumulation in beta cells using an alloxan-induced model for beta cell loss.

## Materials and Methods

### Radiolabeling

[Lys^40^(DTPA)]exendin-3 (Peptides Specialty Laboratories, Heidelberg, Germany) was radiolabeled as previously described [14]. Briefly, 150 MBq [^111^In]InCl_3_ was added to 1 μg [Lys^40^(DTPA)]exendin-3 dissolved in 0.1 M 2-(*N*-morpholino)ethanesulfonic acid (MES), pH 5.5 (Sigma-Aldrich, St. Louis, MO, USA) and incubated for 20 min at room temperature (RT). After incubation, EDTA (Sigma-Aldrich) and Tween 80 (Sigma-Aldrich) were added to a final concentration of 5 mM and 0.1 %, respectively. The radiochemical purity of [^111^In]exendin-3 was determined by instant thin-layer chromatography (ITLC) (ITLC-SG, Agilent Technologies, Lake Forest, CA, USA), using 0.1 M EDTA in 0.1 M NH_4_Ac (Sigma-Aldrich), pH 5.5 as a mobile agent.

### Animals

All animal experiments were approved by the Animal Welfare Committee of the Radboud University, Nijmegen and carried out in accordance with the local and national guidelines. Six- to 8-week-old male animals were used for all experiments. For the experiments in mice, BALB/c, DBA, CBA, and C57Bl/6J mice were purchased from Janvier Labs (Le Genest Saint Isle, France) and the NMRI mice from Harlan (Horst, The Netherlands). For the experiments in rats, Brown Norway, F344, WAG/Rij, and Sprague Dawley rats were purchased from Harlan.

### Alloxan Treatment

To determine the specificity of [^111^In]exendin accumulation in the beta cells, animals were injected with various doses (25–75 mg/kg) of alloxan monohydrate (Sigma Chemicals, St. Louis, MO, USA) to deplete the beta cells. Alloxan was dissolved in ice-cold 10 mM HCl with a concentration of 0.1 mg/μl and diluted with ice-cold phosphate buffered saline (PBS). During storage and dilution, alloxan was protected from light and kept on ice. Animals were injected intravenously with 200 μl alloxan solution within 5 min after dilution. Control animals were injected with vehicle (10 mM HCl diluted with PBS). Blood glucose concentrations were monitored for 1 week after alloxan injection using a blood glucose meter (Accu-Chek Sensor, Roche Diagnostics, Almere, The Netherlands).

### Biodistribution Studies

Biodistribution studies were performed to compare the [^111^In]exendin uptake in different mouse and rat strains. Based on the pancreatic uptake, endocrine-to-exocrine ratio, pancreas-to-stomach ratio, and pancreas-to-duodenum ratio, one strain was chosen to determine the specificity of [^111^In]exendin accumulation in the beta cells, as described above. To determine the effect of alloxan over time, biodistribution studies were performed on day 1, 3, 5, and 7 after alloxan injection. All animals used for biodistribution studies were injected intravenously with 15 MBq [^111^In]exendin-3 (peptide dose 20 pmol/rat, mouse). Non-GLP-1R-mediated exendin uptake was determined in a separate group of animals that were coinjected with an excess of unlabeled exendin (25 μg/animal). Four hours after injection of the radiolabeled exendin, animals were euthanized and the pancreas and other relevant tissues (blood, muscle, heart, lung, spleen, kidney, liver, stomach, and duodenum) were dissected, weighed, and counted in a well-type gamma counter (Wallac 1480 Wizard, Perkin Elmer, Boston, MA, USA). The percentage injected dose per gram tissue (%ID/g) was determined for each tissue.

### Digital Autoradiography

*Ex vivo* autoradiography was performed to visualize the uptake in the islets and the exocrine pancreas. After dissection, pancreata were fixed in 4 % formalin (*w*/*v*), dehydrated, and embedded in paraffin. Sections of the pancreas (4 μm) were prepared and exposed to a phosphorimaging plate (Fuji Film BAS-SR 2025, Raytest, Straubenhardt, Germany) for 7 days. Images were acquired with a radioluminography laser images (Fuji Film BAS 1800 II system (Raytest) and analyzed using Aida Image Analyzer software (Raytest).

### Endocrine-to-Exocrine Ratio Determination

To quantitatively compare tracer uptake in the islets and the exocrine pancreas, endocrine-exocrine uptake ratios were determined in the autoradiographs of pancreatic sections. The digital images were analyzed using ImageJ, a public domain software (http://imagej.nih.gov/ij/). Three pancreatic sections were analyzed per mouse and rat strain. Per pancreatic section, three regions of interest (ROI) were drawn in both the exocrine and endocrine pancreas and the mean density values (photostimulated luminescence) were determined. These values were used to calculate the endocrine-exocrine uptake ratio.

### Immunohistochemistry

Pancreatic sections (4 μm) of C57Bl/6 mice, Brown Norway rats, and humans were stained for the presence of GLP-1R. Antigen retrieval was performed in 10 mM sodium citrate, pH 6.0 for 10 min at 96 °C. Subsequently, sections were incubated for 10 min with 3 % H_2_O_2_ in PBS at RT in the dark, to block endogenous peroxidase activity. Nonspecific binding was blocked by incubation for 30 min with 5 % normal swine serum. The primary anti-GLP-1R antibody (ab39072, Abcam, Cambridge, UK) was diluted in PBS containing 1 % BSA (1:500). Primary antibody incubation for 90 min was followed by incubation with swine-anti-rabbit peroxidase (1:50) (p0271, DAKO, Copenhagen, Denmark) for 30 min at RT in the dark. Finally, 3,3′diaminobenzidine (DAB) was used to develop the pancreatic sections.

### Pancreatic Islet Isolation, mRNA Extraction, and Real-Time PCR

mRNA extraction and real-time PCR was performed as described previously [25]. Briefly, poly(A)+mRNA was isolated from pancreatic islets (rat islets were isolated by collagenase digestion and handpicking and mouse islets using the Histopaque method [26]) or exocrine tissue (the tissue remaining after islet isolation) from C57Bl/6 mice and Wistar rats using the Dynabeads mRNA DIRECT^™^ kit (Invitrogen, Merelbeke, Belgium) and reversely transcribed. Exocrine mRNA samples used in the study were evaluated with Biodrop (Cambridge, UK) and showed a 260/280 ratio >1.9, suggesting that the mRNA was well preserved. Real-time PCR amplification of *Glp-1r* was performed using IQ SyBR Green Supermix on iCycler MyiQ Single Color (BIO-RAD, Hercules, CA, USA) and compared to a standard curve. *amy2* was calculated with the deltaCT method [27]. In all assays, the geometrical means of the housekeeping genes *β-actin* and *gapdh* was used as a reference. The pancreatic islet and exocrine tissue preparations were selected based on the expression levels of, respectively, the endocrine marker *pdx1* and the exocrine marker *amy2*. The primers used are listed in Table 1. Standard curves of the reference genes (*β-actin* and *gapdh*) and *Glp1r* mRNA expression assays of both rat and mouse are provided in ESM Fig. 1.

**Table 1:** Primers used for quantitative PCR on rat and mouse cDNA with the length of amplification on base pairs

Primer	Sequence	Base pairs
Rat GLP-1R RT		
Forward (5′–3′)	GGCTCCTCTCGTATCAGGAC	200
Reverse (5′–3′)	GATAACGAACAGCAGCGGAAC	
Rat GLP-1R std		
Forward (5′–3′)	ATCCACCTGAACCTGTTTGC	468
Reverse (5′–3′)	CTTGGCTATCACGATGCAGA	
Mouse GLP-1R RT		
Forward (5′–3′)	GCGTGGCAGCCAACTACTA	139
Reverse (5′–3′)	ATAACGAACAGCAGCGGAAC	
Mouse GLP-1R std		
Forward (5′–3′)	ATCCACCTGAACCTGTTTGC	617
Reverse (5′–3′)	AGCTTGATGAAGCGTAGGGT	
Rat insulin RT		
Forward (5′–3′)	TGTGGTTCTCACTTGGTGGA	111
Reverse (5′–3′)	CTCCAGTTGTGCCACTTGTG	
Rat insulin std		
Forward (5′–3′)	TGACCAGCTACAGTCGGAA	390
Reverse (5′–3′)	GTTGCAGTAGTTCTCCAGTTGG	
Mouse insulin RT		
Forward (5′–3′)	GGAAGCCCCGGGGACCTTCAGA	138
Reverse (5′–3′)	GGCGGGTCGAGGTGGGCCTTA	
Mouse insulin std		
Forward (5′–3′)	CACCCAAGTCCCGCCGTGAAG	449
Reverse (5′–3′)	TCAGTGGCATTTACACGGTTGCCTA	
β-Actin RT		
Forward (5′–3′)	CTGTACGCCAACACAGTGCT	127
Reverse (5′–3′)	GCTCAGGAGGAGCAATGATC	
β-Actin std		
Forward (5′–3′)	AAATCTGGCACCACACCTTC	805
Reverse (5′–3′)	CCGATCCACACGGAGTACTT	
Rat pdx-1 RT		
Forward (5′–3′)	GGTATACCAGCGAGATGCT	152
Reverse (5′–3′)	TCAGTTGGGAGCCTGATTCT	
Rat pdx-1 std		
Forward (5′–3′)	GAGGACCCGTACAGCCTACA	748
Reverse (5′–3′)	GGGACCGCTCAAGTTTGTAA	
Mouse pdx-1 RT		
Forward (5′–3′)	GAGGTGCTTACACAGCGGAA	116
Reverse (5′–3′)	GGGCCGGGAGATGTATTTGT	
Mouse pdx-1 std		
Forward (5′–3′)	CCTTTCCCGAATGGAACCGA	679
Reverse (5′–3′)	GCTCTCGTGCCCTCAAGAAT	
Rat amy2 RT		
Forward (5′–3′)	ATTGATCTTGGTGGTGAAGCA	174
Reverse (5′–3′)	GGCTCTGTCAGTAGGCACAA	
Rat amy2 std		
Forward (5′–3′)	CGAACCAAGGTGGCTGACTAT	636
Reverse (5′–3′)	TCGATGTTCACAGACCCAGT	
Mouse amy2 RT		
Forward (5′–3′)	CATGGTGACAAGGTGCAACA	102
Reverse (5′–3′)	CAGGTACTGCTTGTTCCTGC	
Mouse amy2 std		
Forward (5′-3′)	TCTGCACAAGGTCTGGAAATGA	500
Reverse (5′–3′)	ACCCAGATCAATGACCTCTTGG	
Gadph RT		
Forward (5′–3′)	GCCTGGAGAAACCTGCCAAGTATGA	101
Reverse (5′–3′)	AACCTGGTCCTCAGTGTAGCCC	
Gadph std		
Forward (5′–3′)	ATGACTCTACCCACGGCAAG	975
Reverse (5–3′)	TGTGAGGGAGATGCTCAGTG	

## Results

### [^111^In]Exendin Uptake in Various Mouse Strains

**Fig. 1:**
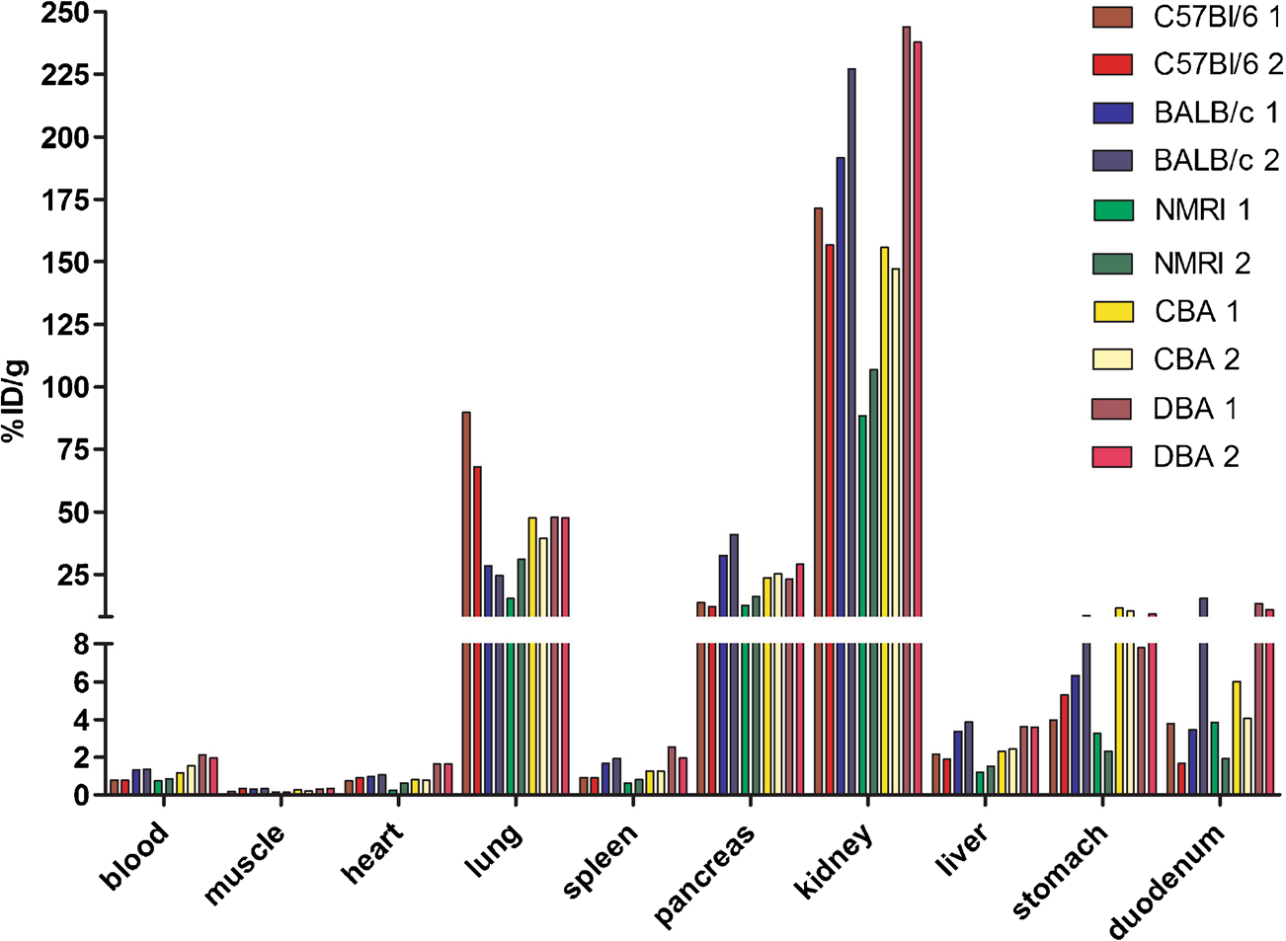
Biodistribution of [^111^In]exendin in C57Bl/6, BALB/c, NMRI, CBA, and DBA mice. Values are expressed as percentage injected dose per gram tissue (*n* = 2). Mice were dissected 4 h after injection.

Figure 1 shows the biodistribution (two mice per strain) of [^111^In]exendin in five mouse strains. As reported previously [28], tracer uptake was observed not only in the pancreas but also in various other organs, such as the lung, stomach, duodenum, and the kidneys. Based on these results, BALB/c mice showed favorable features for *in vivo* beta cell targeting, namely high pancreatic uptake (36.8 %ID/g) and relatively low uptake in lung (26.4 %ID/g), stomach (7.37 %ID/g), and duodenum (9.42 %ID/g). Therefore, BALB/c mice were selected to determine the specificity of tracer accumulation in the beta cells. C57Bl/6 mice, a strain often used in diabetes research, was also evaluated.

### Beta Cell Specificity of [^111^In]Exendin Uptake in BALB/c and C57Bl/6 Mice

**Fig. 2:**
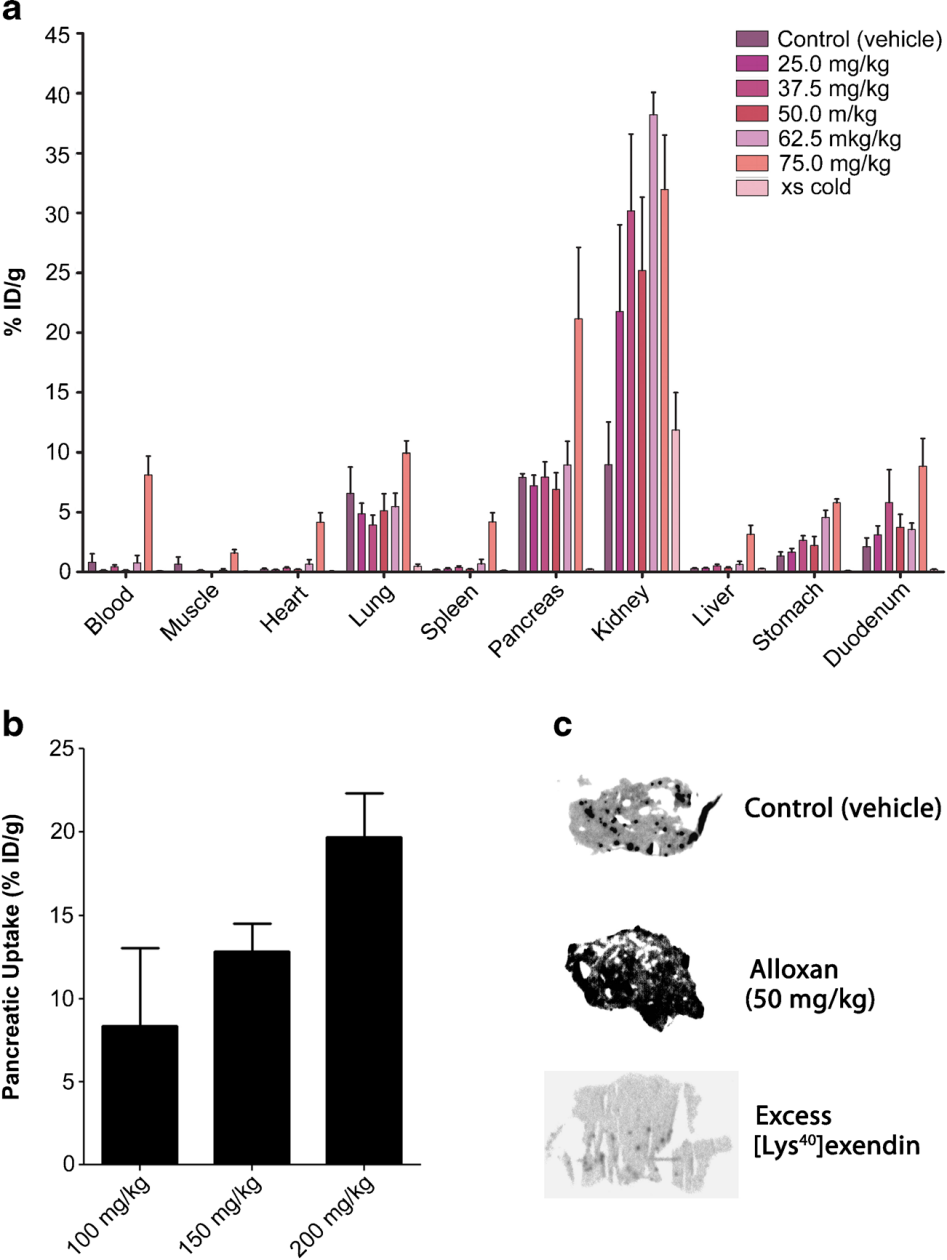
**a** Biodistribution of [^111^In]exendin in control and alloxan-treated BALB/c mice. Values are expressed as percentage injected dose per gram tissue (*n* = 5 mice). Blocking was performed by coinjection of 25 μg unlabeled exendin (*n* = 5). **b** Pancreatic uptake of [^111^In]exendin in BALB/c mice treated with 100 mg/kg (*n* = 5), 150 mg/kg (*n* = 4), and 200 mg/kg (*n* = 3) of alloxan. Values are expressed as percentage injected dose per gram tissue. **c** *Ex vivo* autoradiography of pancreatic sections of BALB/c mice treated with PBS (control) and 50 mg/kg alloxan. Blocking was performed by coinjection of 25 μg unlabeled exendin.

**Fig. 3:**
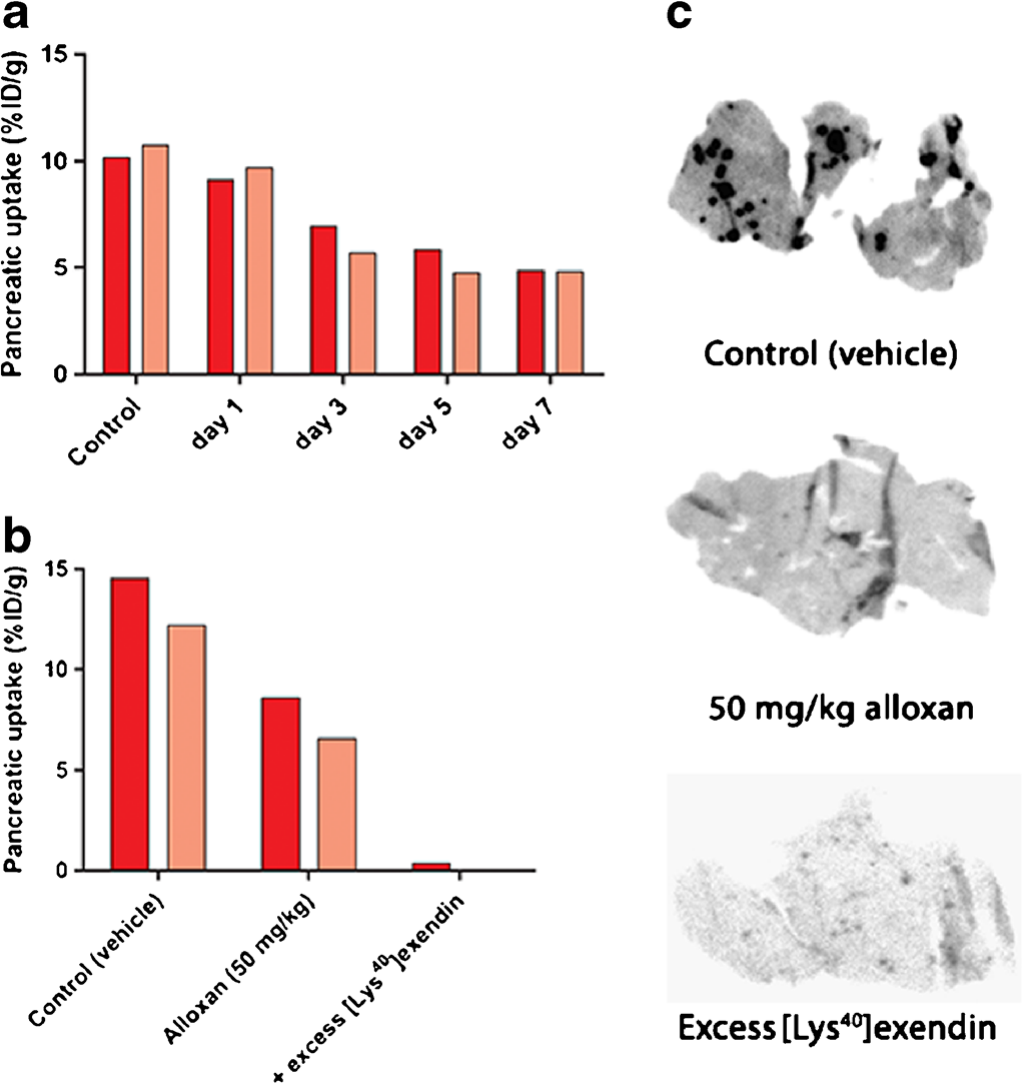
**a** Pancreatic uptake of [^111^In]exendin in C57Bl/6 mice (*n* = 2) on different days after alloxan treatment (50 mg/kg). **b** Pancreatic uptake of [^111^In]exendin in C57Bl/6 mice treated with vehicle (control) (*n* = 2) and 50 mg/kg alloxan (*n* = 2). Blocking was performed by coinjection of 25 μg unlabeled exendin (*n* = 1). **c** Autoradiography of pancreatic sections of C57Bl/6 mice treated with vehicle (control) and 50 mg/kg alloxan. Blocking was performed by coinjection of 25 μg unlabeled exendin.

In BALB/c mice, the pancreatic uptake did not decline after injection of 25, 32.5, 50, and 62.5 mg/kg alloxan (Fig. 2a). Mice treated with even higher doses of alloxan (75, 100, 150, and 200 mg/kg) showed increased total pancreatic tracer uptake. In animals treated with high alloxan doses, elevated tracer levels in the blood were observed and thus, higher uptake in all other tissues, including the exocrine pancreas (Fig. 2a, b). This is most probably the result of dehydration secondary to severe hyperglycemia, leading to slower blood clearance. In addition, direct toxic effects of alloxan cannot be excluded at high doses. Coinjection of an excess unlabeled exendin decreased the pancreatic uptake from 7.9 ± 0.6 %ID/g to 0.23 ± 0.04 %ID/g (Fig. 2a) which was also reflected by the autoradiographic images (Fig. 2c). Due to the high uptake in exocrine tissue, the endocrine-exocrine ratio in these mice was only 4.11 ± 0.93 (Table 2) which is very low when compared to rats (see below). These results—especially the reduced uptake after coinjection of unlabeled exendin—indicate specific, receptor-mediated tracer binding in the exocrine pancreas of BALB/c mice. Because of this high exocrine uptake, [^111^In]exendin uptake after alloxan treatment was further investigated in C57Bl/6 mice for validation of the results described above. Figure 3a summarizes pancreatic uptake of [^111^In]exendin in C57Bl/6 mice on various days after alloxan treatment. After 3 days, a clear decrease in pancreatic uptake was observed, while the maximum decrease was observed 7 days after alloxan treatment. However, the decrease did not exceed 40 % compared to control mice injected with vehicle. Furthermore, similar to the findings observed in BALB/c mice, tracer uptake in the exocrine pancreas of C57Bl/6 mice could be blocked with an excess of unlabeled exendin (Fig. 3b, c). Additionally, the calculated endocrine-to-exocrine uptake ratio in these mice (4.56 ± 0.91) (Table 2) was in the same range as the ratio observed in BALB/c mice.

**Table 2:** Endocrine-exocrine ratio of [^111^In]exendin uptake in BALB/c and C57Bl/6 mice calculated from autoradiography of pancreatic sections

Mouse strain	Endocrine-exocrine ratio
BALB/c	4.11 ± 0.93
C57Bl/6	4.56 ± 0.91

### [^111^In]Exendin Uptake in Different Rat Strains

**Fig. 4:**
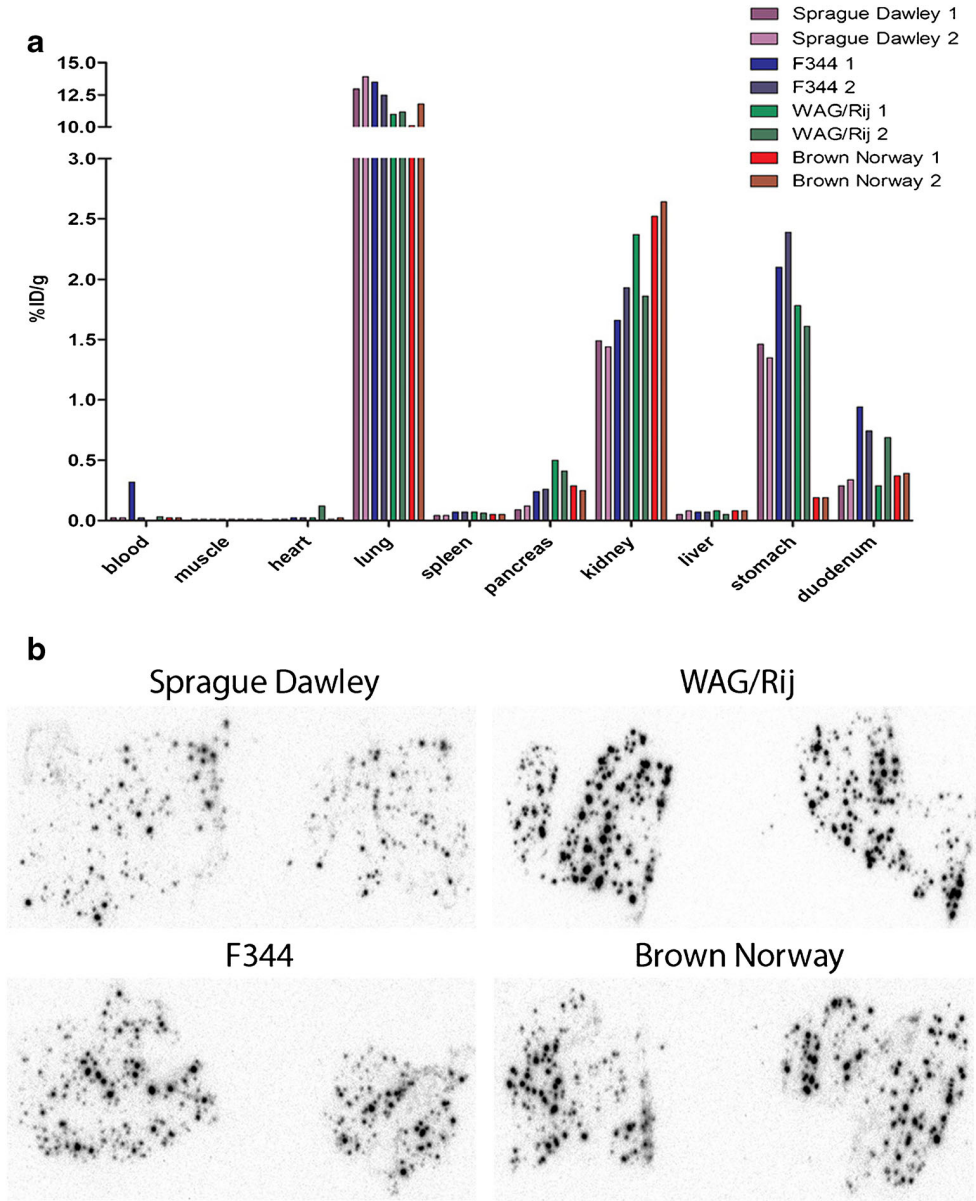
**a** Biodistribution of [^111^In]exendin in Sprague Dawley, F344, Wag/Rij, and Brown Norway rats. Values are expressed as percentage injected dose per gram tissue (*n* = 2 rats). Rats were dissected 4 h after injection. **b** Autoradiography of pancreatic section of Sprague Dawley, F344, Wag/Rij, and Brown Norway rats showing focal hotspots of tracer accumulation representing the islets.

Figure 4a summarizes the biodistribution (two rats per strain) of [^111^In]exendin in four rat strains. In all rat strains, there was very high lung uptake (>10 %ID/g), which was even higher than the uptake observed in the kidneys. The biodistribution profiles indicated that WAG/Rij rats have the highest pancreatic [^111^In]exendin uptake (0.45 %ID/g). In addition, the endocrine-to-exocrine uptake ratio, calculated from the autoradiographs shown in Fig. 4b, was highest for WAG/Rij rats as well (Table 3). The uptake in the stomach (1.7 %ID/g) of WAG/Rij rats is relatively high (Fig. 4a), resulting in a pancreas-to-stomach uptake ratio of 0.27 ± 0.02 (Table 4). Since high stomach uptake might hamper BCM visualization *in vivo*, we selected Brown Norway rats to investigate the beta cell specificity of pancreatic [^111^In]exendin accumulation due to their higher pancreas-to-stomach (1.39 ± 0.11) and acceptable pancreas-to-duodenum (0.70 ± 0.1) ratios (Table 4) despite their lower pancreatic uptake (0.27 %ID/g) (Fig. 4a) and endocrine-to-exocrine ratio (57.1 ± 12.5) (Table 3).

**Table 3:** Overview of the endocrine-exocrine ratios of [^111^In]exendin uptake in various rat strains calculated from autoradiography of pancreatic sections

Rat strain	Endocrine-exocrine ratio
WAG/Rij	105.66 ± 29.84
Brown Norway	57.06 ± 12.53
Sprague Dawley	44.00 ± 13.33
F344	44.83 ± 2.85

**Table 4:** Overview of the pancreas-to-stomach and pancreas-to-duodenum ratios of [^111^In]exendin uptake in the different rat strains

Rat strain	Pancreas-to-stomach ratio	Pancreas-to-duodenum ratio
Sprague Dawley	0.08 ± 0.02	0.33 ± 0.02
F344	0.11 ± 0.00	0.31 ± 0.07
Wag/Rij	0.27 ± 0.02	1.15 ± 0.78
Brown Norway	1.39 ± 0.11	0.70 ± 0.10

### Beta Cell Specificity of [^111^In]Exendin Uptake in Brown Norway Rats

**Fig. 5:**
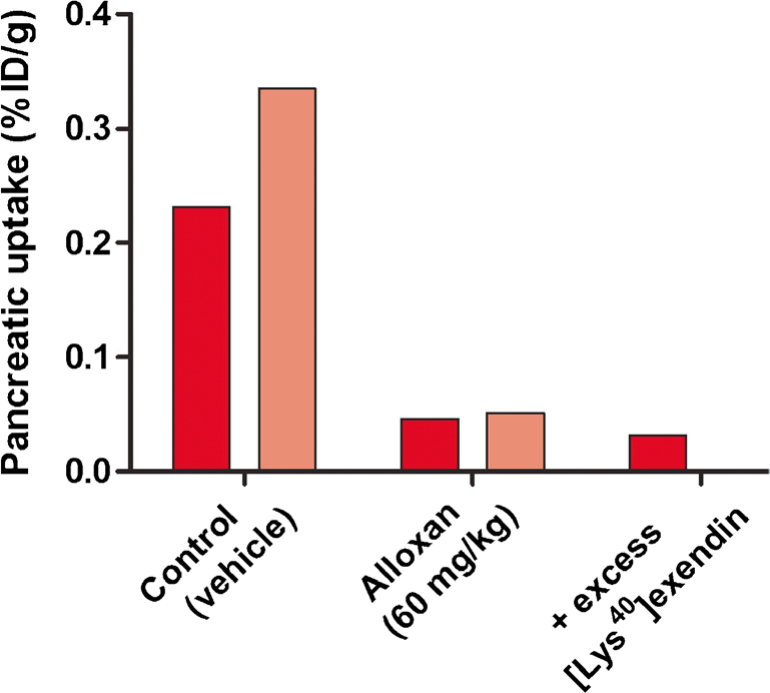
Pancreatic uptake of [^111^In]exendin in Brown Norway rats treated with vehicle (control) (*n* = 2) and alloxan (60 mg/kg) (*n* = 2). Values are expressed as percentage injected dose per gram tissue. Blocking was performed by coinjection of 25 μg unlabeled exendin (*n* = 1).

In Brown Norway rats, treated with 60 mg/kg alloxan (two rats), pancreatic uptake was reduced by more than 80 % compared to control rats, that were injected with vehicle (PBS) (two rats) (Fig. 5). Coadministration of an excess unlabeled exendin (one rat) reduced the pancreatic uptake to similar uptake levels observed in alloxan-treated rats, suggesting no or very low nonspecific [^111^In]exendin uptake in the exocrine pancreas. Furthermore, autoradiographical analysis showed similar results: high, specific tracer uptake in the islets of Langerhans and low, nonspecific uptake in the exocrine pancreas (Fig. 4b) resulting in an endocrine-to-exocrine uptake ratio of 57.1 ± 12.5 (Table 4). These results indicate high beta cell specificity of [^111^In]exendin in these rats.

### Quantitative PCR

**Fig. 6:**
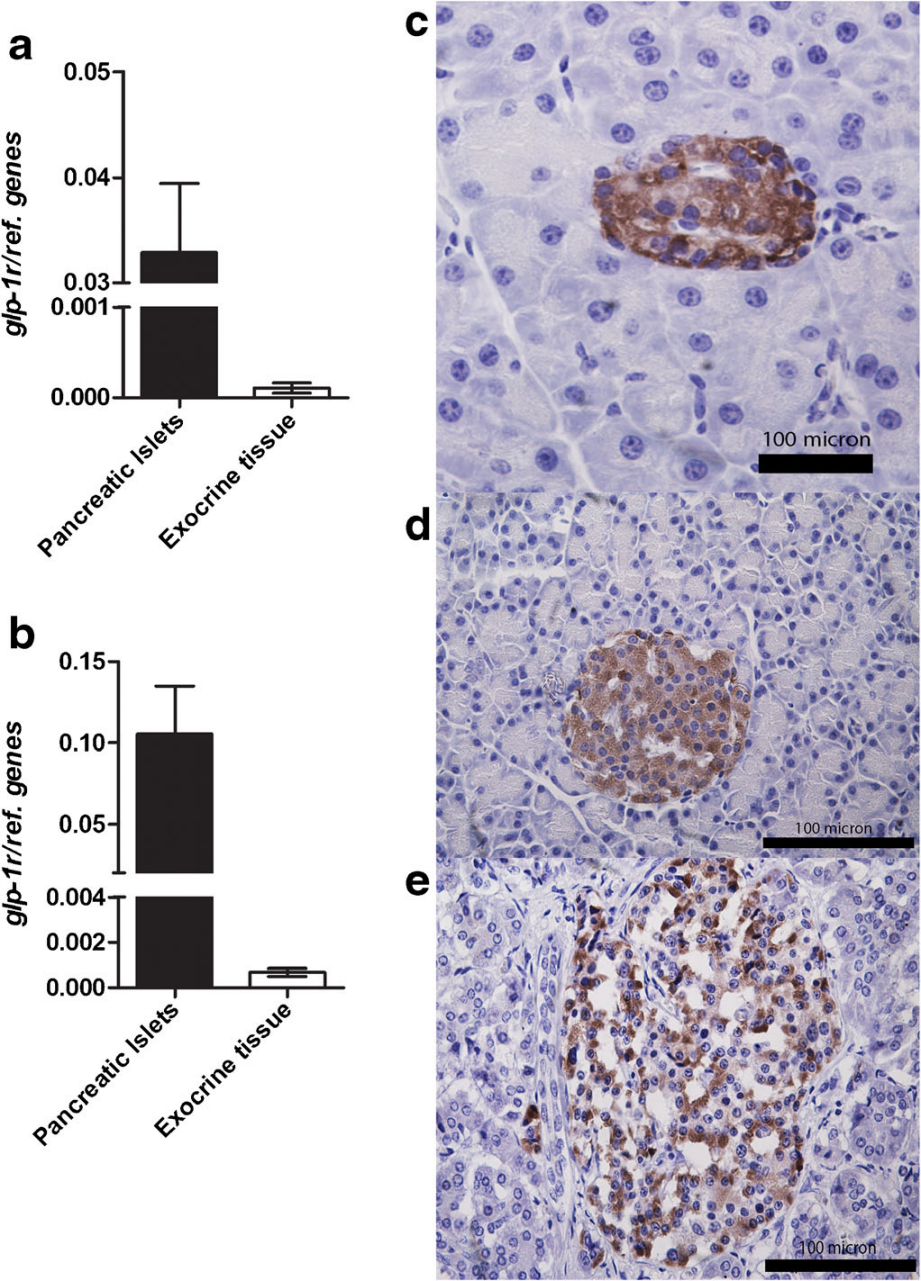
Quantitative PCR for the *Glp-1r* in endocrine and exocrine pancreatic tissue of **a** rat and **b** mouse and immunohistochemical analysis of GLP-1R expression in pancreatic tissue (embedded in paraffin) of **c** rat, **d** mouse, and **e** human. Both analyses show high GLP-1R expression in the islets and no, or very low expression in exocrine tissue. For quantitative PCR, paired *t* test was performed and *p* < 0.05 was considered as significant.

Since receptor-mediated [^111^In]exendin uptake was observed in exocrine pancreas of mice, *Glp-1r* mRNA expression was compared in endocrine and exocrine pancreatic tissue of rats and mice. Quantitative RT-PCR revealed similar *Glp-1r* mRNA expression levels in endocrine and exocrine tissue of rat and mouse (Fig. 6a, b) with endocrine-to-exocrine ratios of 45.8 and 55.6, respectively. Individual expression levels of all cell markers (*Glp-1r*, *Pdx1*, *Ins*, and *Amy2b*) in endocrine and exocrine tissue are provided in ESM Fig. 2.

### Immunohistochemistry

To investigate GLP-1R expression in endocrine and exocrine pancreatic tissue, GLP-1R staining was performed on rat, mouse, and human pancreatic tissue. Figure 6c–e shows the results of the immunohistochemical analysis. In all pancreatic tissues analyzed, the islets of Langerhans showed clear GLP-1R staining while no, or very limited, staining was observed in the exocrine portion of the pancreatic tissue.

## Discussion

In this study, we assessed differences between rodent models for noninvasive *in vivo* BCM assessment via GLP-1R targeting. We investigated the biodistribution of [^111^In]exendin in various rat and mouse strains and determined the specificity of tracer accumulation in the beta cells. Our present findings indicate that Brown Norway rats display the most favorable characteristics for noninvasive BCM assessment using radiolabeled exendin as a tracer. Mice seem to display receptor-mediated tracer uptake in the exocrine pancreas, an observation explaining the limited reduction of exendin uptake in the pancreas of mouse models after beta cell destruction [22, 24].

In view of the spatial resolution of present imaging modalities and the small size of the islets, preventing visualization of single islets *in vivo*, the optimal model for *in vivo* BCM determination must display high and specific [^111^In]exendin accumulation in the endocrine pancreas [29, 30] with no or low uptake in the exocrine pancreas and surrounding tissues, in particular stomach and duodenum that are localized in the vicinity of the pancreas. Since [^111^In]exendin is excreted via the kidneys, high tracer uptake is observed in this organ, which renders accurate quantification of pancreatic tracer uptake challenging in rodents. However, spillover signal from the kidneys can be excluded by analyzing a particular region of interest in the pancreas localized cranial and anterior of the kidneys [14]. Another option to overcome spillover effects of the kidneys is coinjection of a second radiotracer, specifically targeting the exocrine tissue such as [^99m^Tc]demobesin and L-[^123^I]iodophenylalanine in combination with [^111^In]exendin, which facilitates exact delineation of the pancreas and therefore improves accurate quantification of pancreatic [^111^In]exendin uptake [22]. Of all investigated rat strains, WAG/Rij rats showed the highest pancreatic [^111^In]exendin uptake. However, due to their less favorable pancreas-to-stomach and pancreas-to duodenum ratios, WAG/Rij rats are expected to be less suited for *in vivo* BCM determination using [^111^In]exendin SPECT than Brown Norway rats.

To evaluate the beta cell specificity of the tracer, *ex vivo* autoradiography was performed. In rats, focal hotspots of tracer accumulation, representing the beta cells, were observed while there was a low background signal in the exocrine tissue (Fig. 4b). Beta cell depletion by alloxan injection resulted in disappearance of the tracer accumulating hotspots, confirming beta cell specificity of GLP-1R targeting using [^111^In]exendin allowing accurate BCM determination of BCM. Based on autoradiographic analysis, we showed endocrine-to-exocrine ratios of 105.7 ± 29.8 in WAG/Rij rats, 57.1 ± 12.5 in Brown Norway rats, 44.0 ± 13.3 in Sprague Dawley rats, and 44.8 ± 2.9 in F344 rats, which is in line with previous observations [23]. Furthermore, pancreatic [^111^In]exendin uptake in Brown Norway rats is similarly decreased after alloxan-induced beta cell depletion and coinjection with an excess unlabeled exendin, confirming beta-cell-specific tracer accumulation in this model. We have previously shown clear differences in [^111^In]exendin uptake in the pancreas of Brown Norway rats treated with various concentrations of alloxan, resulting in a linear correlation of SPECT signal and BCM [14]. These observations confirm that this rat strain provides an optimal model for *in vivo* BCM assessment via GLP-1R targeting.

Several groups have reported a decrease in pancreatic tracer uptake in mice after alloxan or streptozotocin-induced beta cell destruction [22, 24]. However, the maximum decrease did not exceed 40 %, an observation which can be explained by our data. After coinjection of an excess unlabeled exendin in mice, tracer uptake was much lower than the remaining uptake observed after alloxan-induced beta cell depletion. These results strongly suggest that the limited decrease in tracer uptake after alloxan-induced beta cell destruction is caused by receptor-mediated [^111^In]exendin uptake in the exocrine pancreas of mice. Nevertheless, there was no detectable GLP-1R expression in exocrine pancreatic tissue of mice by immunohistochemistry and only very low *Glp-1r* mRNA expression was observed by RT-PCR. Furthermore, endocrine-to-exocrine ratios of *Glp-1r* mRNA expression are similar in mice and rats. These observations suggest that [^111^In]exendin probably binds to a receptor other than the GLP-1R in the exocrine pancreatic tissue of mice, which could also explain the observations of Nalin et al. [31]. These findings preclude the use of this model for additional characterization of [^111^In]exendin uptake. In contrast with observations in mice after beta cell destruction [22, 24] (present data), and similar to the above described rat data, patients with long standing type 1 diabetes can show very low [^111^In]exendin uptake similar to background values [14], suggesting no or negligible uptake in the exocrine pancreas. Therefore, the situation in humans appears to be better reflected by rat models than by mouse models. At this point in time, it remains unclear which receptor is responsible for exendin uptake in the exocrine pancreas of mice but it is highly likely that this receptor shows differences with the rat and human receptors in a way that only the variant expressed in mice can bind exendin.

## Conclusion

The choice of a certain rodent model can greatly influence studies investigating BCM dynamics. The present findings indicate that mice have binding in the exocrine pancreas which is mediated by a receptor that is not the GLP-1R, rendering them an inadequate model for BCM assessment by radiolabeled exendin. On the other hand, rats do not show receptor-mediated exendin binding in the exocrine pancreas, and are therefore a better suited model for further studies. Among the investigated rat models, Brown Norway rats are the optimal model for noninvasive GLP-1R targeting given their favorable pancreas-to-background uptake ratios. In view of these results and the very low remaining uptake in patients with T1D, rats appear to display better characteristics for *in vivo* investigation of BCM dynamics when compared to mice, and are thus considered a suitable model better reflecting the human situation than other animal models.

## Electronic supplementary material

Below is the link to the electronic supplementary material.

ESM 1 (PDF 426 kb)
