# Supplementary material for: Strain Differences Determine the Suitability of Animal Models for Noninvasive In Vivo Beta Cell Mass Determination with Radiolabeled Exendin
Source: Mol Imaging Biol. 2016 Feb 17;18(5):705–14. doi: 10.1007/s11307-016-0936-y (PMC5010585; doi:10.1007/s11307-016-0936-y)
Supplement: Supplementary file 1 — (PDF 426 kb) [file 11307_2016_936_MOESM1_ESM.pdf]

Electronic Supplementary Material

Strain Differences Determine the Suitability of Animal Models for Non-Invasive *In Vivo* Beta Cell Mass Determination with Radiolabeled Exendin.

Journal: Molecular Imaging and Biology

[1]Stefanie M.A. Willekens, [1]Lieke Joosten, [1]Otto C. Boerman, [2]Alexander Balhuizen, [2]Decio L. Eizirik, [1]Martin Gotthardt, [1]Maarten Brom

[1]Department of Radiology and Nuclear Medicine, Radboud university medical center, Nijmegen, The Netherlands; [2]ULB Center for Diabetes Research, Université Libre de Bruxelles (ULB), Brussels, Belgium

Corresponding Author: Stefanie Willekens

Department of Radiology and Nuclear Medicine

Radboud university medical center

PO BOX 9101, 6500 HB Nijmegen, The Netherlands

Telephone/fax: +31 24 36 19097 / +31 24 36 18942

Email: Stefanie.willekens@radboudumc.nl

Journal: Molecular Imaging and Biology

Supplemental figures

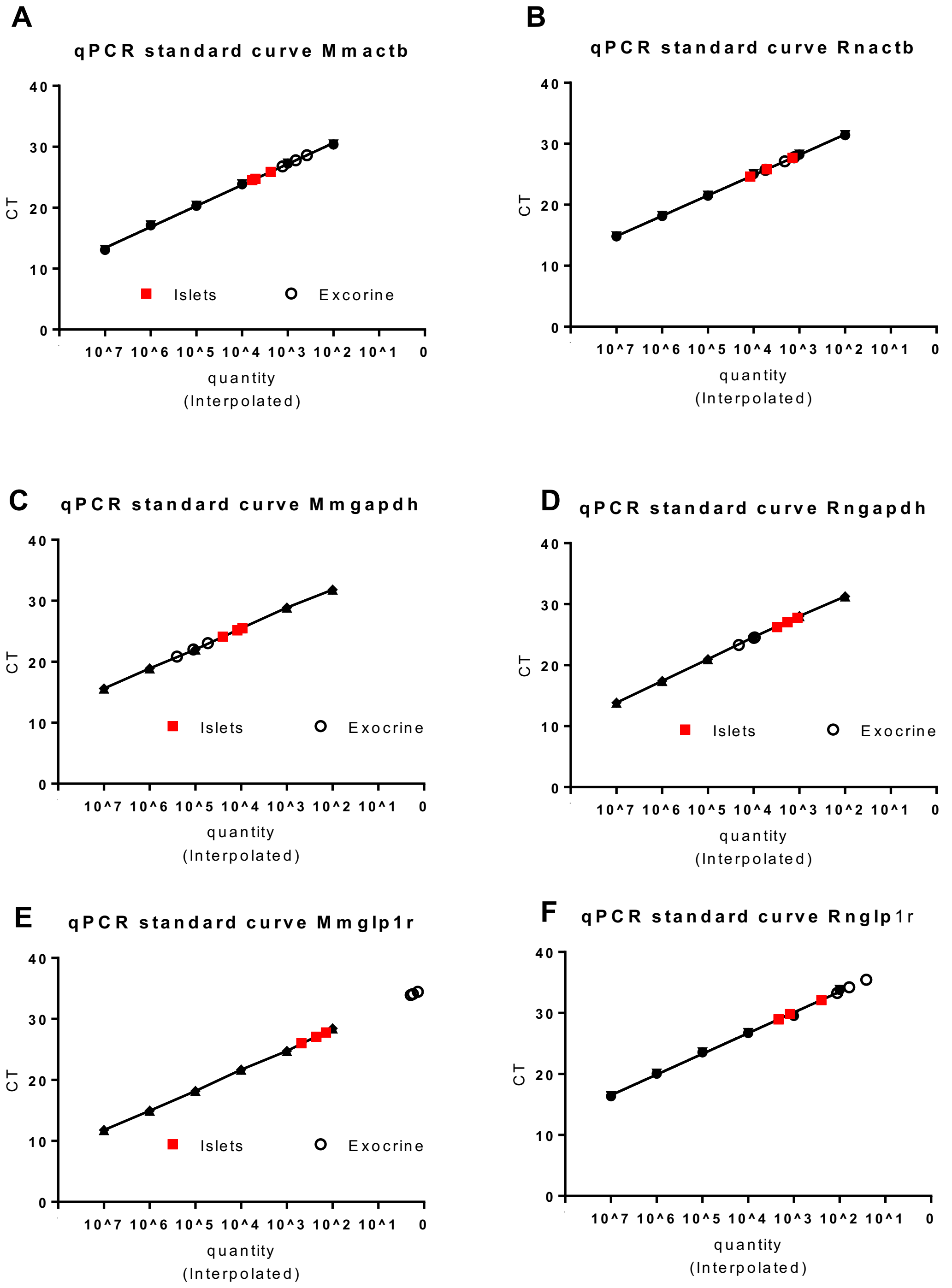

ESM Figure 1: mRNA estimation of glucagon-like peptide 1 receptor (*Glp-1r*) expression was performed by quantitative PCR where beta actin (actb) and glyceraldehyde-3-phosphate dehydrogenase (gapdh) were used as reference genes. Standard curves of *actb*, *gapdh* and *Glp-1r* genes for both mouse (Mm, Mouse musculus, A, C, E) and rat (Rn, Rattus norvegicus, B, D, F) (mRNA expression assays, with plotted CT values of pancreatic islets samples (red squares) and exocrine tissue (black circles).

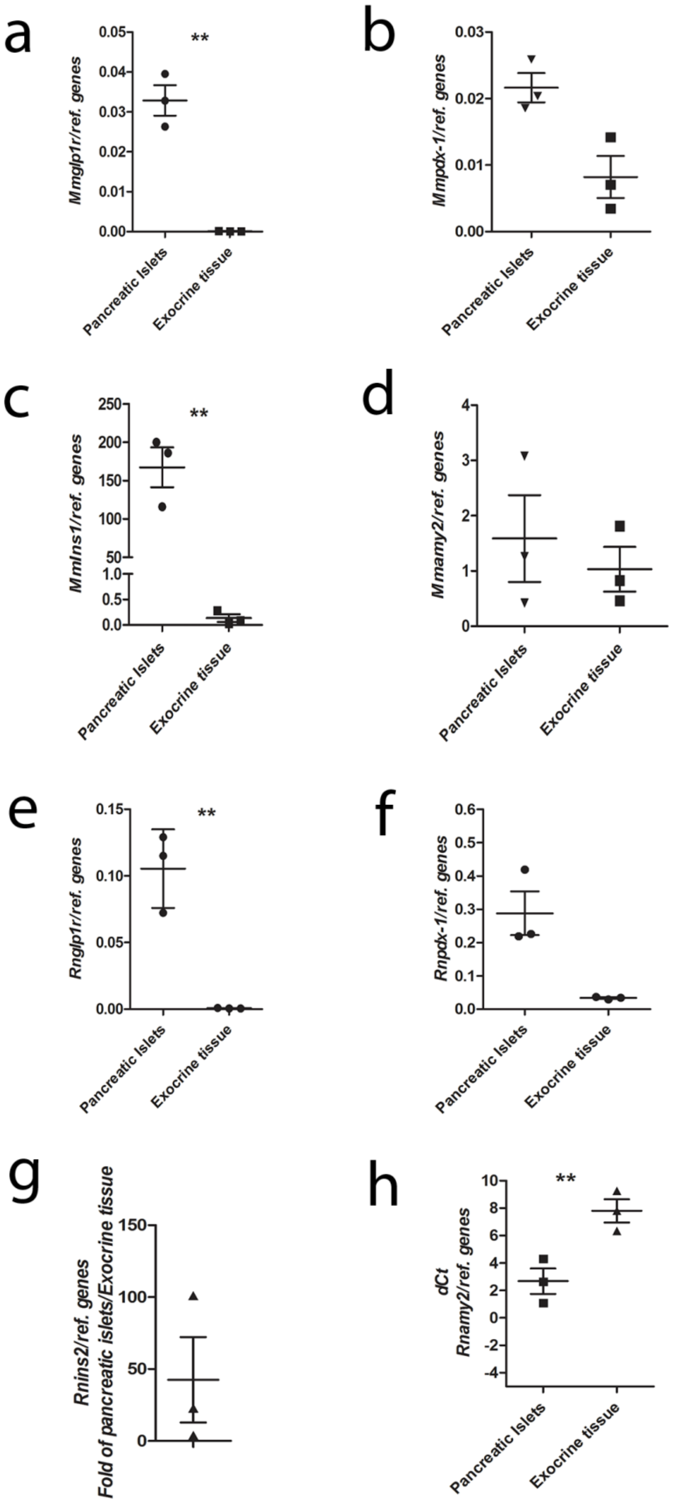

ESM Figure 2: Quantitative PCR of the glucagon-like peptide 1 receptor (*Glp-1r*) mRNA expression in pancreatic islets and exocrine tissue with additional pancreatic endocrine and exocrine cell markers for mouse (Mm, Mouse musculus); A) *Glp-1r*, B) *Pdx1* (Pancreatic and duodenal homeobox 1), and C) *Ins1* (insulin) and D) *Amy2b* (pancreatic α-Amylase), and for rat (Rn, Rattus novergicus); E) *Glp1r*, F) *Pdx1*, G) *Ins2* (Insulin) and H) *Amy2b*. In both species the pancreatic islets were positive for *Glp-1r* and the endocrine cell markers *Pdx1* and *Ins*, while the exocrine material was positive for the exocrine cell marker *Amy2b* but had a decreased expression of *Glp-1r, Pdx1* and *Ins*. N=3 in all experimental conditions. Paired t-test was performed and p<0.05 was considered significant (**).
